# Improving Geometric Uniformity in Dynamic Chemical Vapor Deposition of Carbon Nanotube Forests

**DOI:** 10.1021/acs.iecr.4c03787

**Published:** 2025-05-27

**Authors:** Golnaz Najaf Tomaraei, Moataz Abdulhafez, Soumalya Ghosh, Jaegeun Lee, Mostafa Bedewy

**Affiliations:** † Department of Mechanical Engineering and Materials Science, 6614 University of Pittsburgh, 3700 O’Hara Street, Pittsburgh Pennsylvania 15261, United States; ‡ Department of Industrial Engineering, University of Pittsburgh, 3700 O’Hara Street, Pittsburgh Pennsylvania 15261, United States; § School of Chemical Engineering, 34996 Pusan National University, 2, Busandaehak-ro 63 beon-gil, Geumjeong-gu, Busan 46241, Republic of Korea; ∥ Department of Chemical and Petroleum Engineering, University of Pittsburgh, 3700 O’Hara Street, Pittsburgh Pennsylvania 15261, United States

## Abstract

Geometric nonuniformities are often observed in the catalytic chemical vapor deposition (CVD) of vertically aligned carbon nanotubes (VACNTs), known as CNT forests. These nonuniformities typically occur in the form of sloped heights and empty regions within the catalyst-covered substrate. To realize the true potential of carbon nanotube forests in unidirectional mass and energy transport applications, it is critical to develop robust manufacturing processes to produce geometrically uniform CNT forests on large-scale substrates in a repeatable manner. Our custom-designed reactor with an IR heating multizone furnace with rapid thermal processing capability allows the programming of dynamic recipes with the catalyst formation temperature decoupled from the CNT nucleation and growth temperature. In this work, we present an approach for tuning the geometric uniformity of CNT forests based on the combined effects of substrate holder design and dynamic recipes during CVD. We propose a mechanism that explains the observed geometric nonuniformities based on both the temperature profile across the catalyst chip, which depends on the substrate holder design, and the temperature range for CNT growth, which depends on the catalyst formation temperature. We performed a comparative study of the properties of alumina layers after annealing with two different substrate holder designs. We found that the actual temperature experienced by the sample depends on the substrate holder, which supports our proposed mechanism. Our work provides insight into the growth of CNT forests with large-scale spatial uniformity and high structural quality.

## Graphical abstract

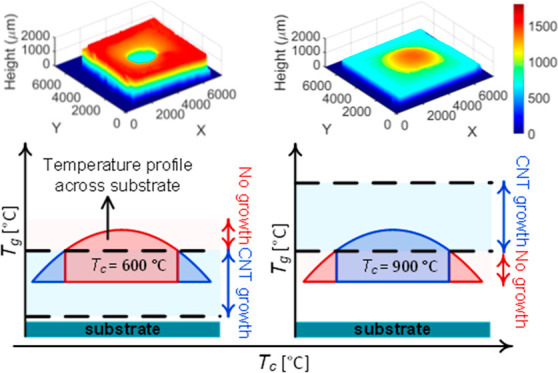

## 1 Introduction

Carbon nanotubes (CNTs) are highly anisotropic structures with high aspect ratios that are typically grown to macroscopic lengths. Individual CNTs possess excellent mechanical strength as well as thermal and electrical conductivity. Importantly, large-scale mass production of highly aligned CNT ensembles in the form of vertically aligned CNT forests is sought after. The interactions and self-organization among a large population of individual CNTs create a forest morphology with high density and high level of alignment. The high anisotropy of the collective properties of CNT forests is essential for successful application as thermal interfaces,^1^ interconnects,^2^ and flow membranes.^3^ Therefore, achieving a uniform height and areal density across the forest during growth is an important, yet challenging requirement.

Catalytic chemical vapor deposition (CVD) is a common method for the synthesis of CNT forests in high yield. Alumina-supported iron nanoparticles are commonly used as substrate-bound catalyst systems for CNT synthesis. In the first step of the process, the catalyst thin film transforms into catalyst nanoparticles through solid-state dewetting in a reducing environment at a high temperature, known as the annealing temperature or the catalyst formation temperature. Upon the introduction of gaseous hydrocarbons, active species from thermally decomposed carbon feedstock react with catalyst nanoparticles, leading to the nucleation and growth of CNTs from active nanoparticles. While most of the research on CNT forests grown by CVD is focused on controlling diameter and number of walls as well as improving yield and density, there are few studies on the large-scale synthesis of CNT forests.^4–7^ Researchers have previously tried to achieve large-scale production of uniform CNT forests via CO_2_-assisted CVD,^8^ controlling the gas flow direction,^7^ and by controlling the growth parameters such that the bulk diffusion of gaseous carbon precursor is the dominant growth regime and the formation of byproducts is suppressed.^4^ While these approaches have been successful in improving large-scale uniformity, they all require additional steps.

There is an abundance of previous studies on using different types of CVD reactors for CNT growth in order to reveal the independent effects of temperature on gas-phase reactions, catalyst reduction, nanoparticle formation by dewetting, and CNT nucleation.^9–19^ We have previously shown the capability of our custom-designed rapid thermal CVD in decoupling the gas-phase decomposition temperature, *T*_p_, catalyst formation temperature, *T*_c_, and CNT nucleation and growth temperature, *T*_g_.^20–23^ We found that while the growth temperature, *T*_g_, primarily affects growth kinetics and the nucleation density of CNTs,^22^ catalyst treatment temperature, *T*_c_, primarily affects the catalytic lifetime,^23^ and the preheater temperature, *T*_p_, can boost the growth yield owing to thermal decomposition of ethylene. However, there is a lack of systematic studies that reveal the dependence of CNT forest uniformity on any aspects of reactor design, especially for cold-walled reactors such as ours, in which substrate heating is achieved by local photothermal interactions.

While characterization and control of CNT forest uniformity has been studied using different imaging techniques,^6,24,25^ our approach of using 3D optical profilometry of centimeter-scale structures uniquely enables accurate spatial mapping of growth. Here, we show that the uniformity of CNT forests is significantly affected by modifying the design of the substrate holder on which the catalyst chip is placed in the rapid thermal CVD reactors. The hypothesis we test and confirm in the present study is that alterations to the substrate holder’s design, material, or surface coating shift the temperature distribution across the catalyst-coated silicon chip and hence influences the geometric uniformity of the CNT forest in reactors equipped with an infrared (IR) source of irradiation and quartz walls. When this facile modification of the substrate holder is combined with the capability of our reactor to allow independent control of *T*_c_ and *T*_g_, we show the successful growth of high-quality CNTs with uniform geometry on chips of approximately 100 mm^2^ area. Our approach offers an effective means to enhance forest uniformity in cold-walled reactors without the need for introducing additional gases or making significant alterations to the reactor’s walls.

By demonstrating the successful growth of high-quality CNTs with uniform macroscopic geometry, our work contributes to the broader goal of large-scale manufacturing of uniform functional CNT-based interfacial materials, coatings, and composites. The findings presented here have implications for various applications in thermal management, interconnects in 3D electronics, and filtration systems, where the performance and reliability critically rely on the uniformity and precise control of the CNT forest geometry and properties on a wafer scale.

## 2 Experimental Section

### 2.1 CNT Forest Growth

CNTs were grown on two types of substrate-bound catalysts. The substrate was a silicon (100) wafer with a 300 nm thick SiO_2_ layer. Each catalyst type consists of a 10 nm thick alumina support layer on a Si wafer, with a 1 nm-thick iron catalyst layer on top. The first type, referred to as Catalyst1, was prepared using atomic layer deposition (ALD) for alumina and e-beam evaporation for iron deposition. In contrast, the second type, referred to as Catalyst2, was fabricated by sputtering both the alumina and iron layers. Both types were cut into various sizes, and the catalyst chips were subsequently loaded into the custom-designed CVD reactor (CVD Equipment, Central Islip, NY), as shown in Figure 1a. The details of the reactor have been described in our previous publications.^20–23,26^ As shown in Figure 1b,c, we utilized two types of holders to confine the movements of the catalyst chips on the quartz tray placed on the paddle inside the rapid thermal processing (RTP) furnace. Holder1 (Figure 1b) consists of two pieces of a 3 in. Si wafer, and Holder2 (Figure 1c) comprises a 2 in. Si wafer with a 50 μm deep recessed area measuring10 mm ×10 mm at the center. A summary of the catalyst types and holder descriptions is given in Table 1.

**Figure 1:**
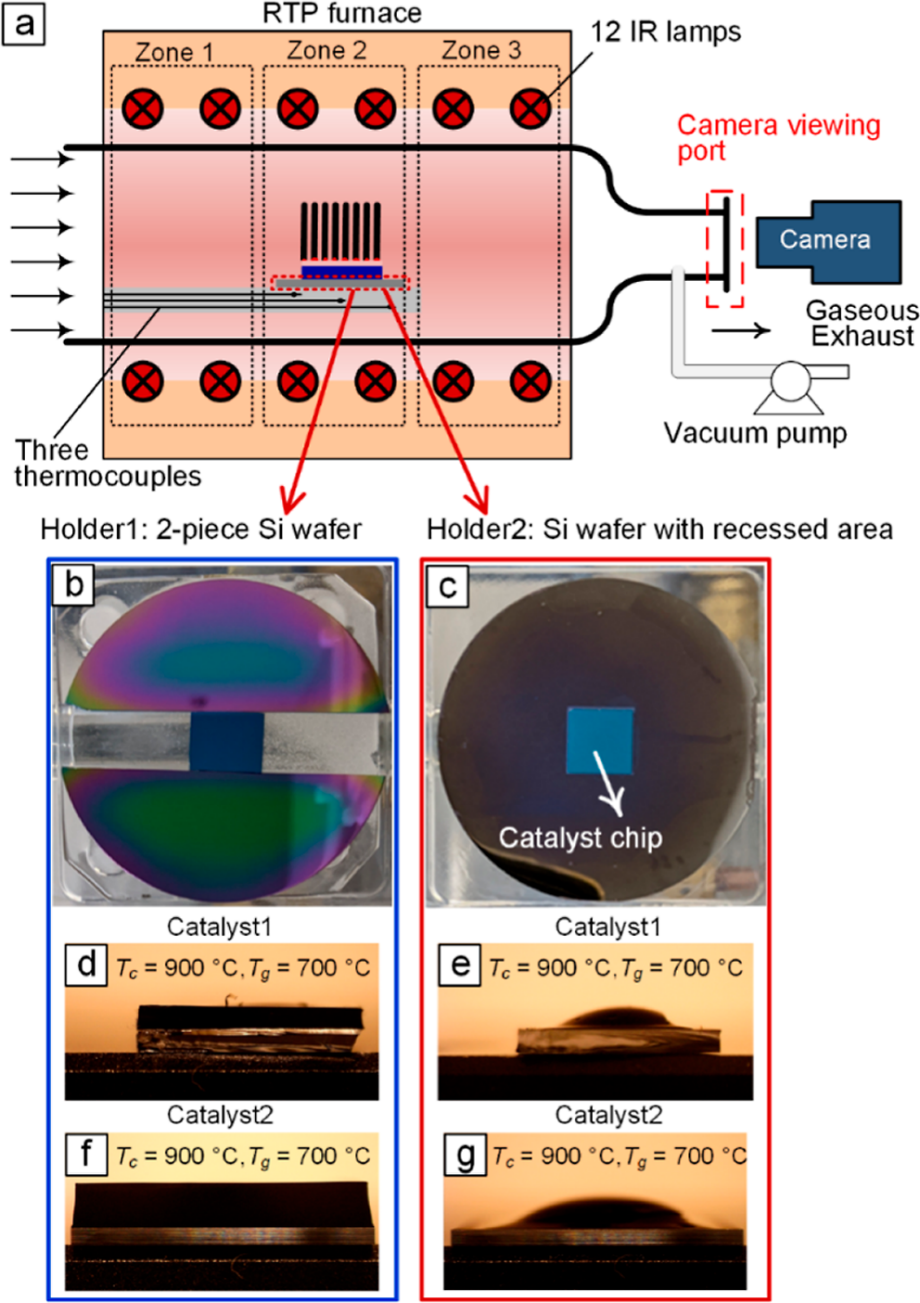
(a) Schematic representation of the multizone RTP furnace in the CVD reactor. Real images of the substrate holders with catalyst chips: (b) Holder1 consisting of two pieces of a 3-in. Si wafer and (c) Holder2, a 2-in. Si wafer with a 10 mm × 10 mm recessed area at the center. Comparison of spatial uniformity between forests grown using these two holders under identical recipes with *T*_c_ = 900 °C and *T*_*g*_ = 700 °C is shown in (d,e). Furthermore, (f,g) demonstrate a similar difference in spatial uniformity when a different catalyst chip is used. Catalyst1 is prepared using ALD for alumina and e-beam evaporation for Fe, while Catalyst2 is prepared through sputtering for both layers.

**Table 1:** Summary of Catalyst Types and Holder Descriptions

catalyst type	catalyst preparation method	holder type	holder description
Catalyst1	ALD for alumina and e-beam evaporation for iron	Holder1	two pieces of 3-in. Si wafer
Catalyst2	sputtering for both alumina and iron	Holder2	2-in. Si wafer with a 50 μm deep recessed areas (10 mm × 10 mm)

The automated growth recipes were programmed by leveraging the capability of our CVD reactor to decouple the gas phase decomposition temperature (*T*_p_), catalyst formation temperature (*T*_c_), and the temperature of CNT nucleation and growth (*T*_g_). In all experiments, a total gas flow rate of 1700 sccm was maintained during both catalyst formation and CNT growth steps. For the catalyst formation step, a hydrogen (H_2_) flow rate of 490 sccm was used to create a reducing environment, and a portion of helium (He) gas flew through a bubbler to generate 100 sccm of water vapor. During CNT growth, 370 sccm of the background He gas was replaced with ethylene, which serves as the carbon feedstock. In the experiments where the gaseous precursor was preheated before entering the RTP furnace, the preheater temperature was set to 825 °C.

### 2.2 Characterization of CNTs

A 3D optical microscope (VR-3000 series, Keyence) was used to study the geometric uniformity of the CNT forests. Additionally, top-view and side-view optical imaging was done to provide a comprehensive analysis of the macroscopic geometry of the forests. Scanning electron microscopy (SEM) imaging was conducted using a Zeiss SIGMA 500 VP instrument to examine the morphology of the as-grown CNT forests, particularly in regions where there is a transition from no or poor growth to the successful growth of vertically aligned CNTs. The structural quality of the CNTs was investigated by Raman spectroscopy (XploRA, Horiba Scientific) using a 638 nm excitation wavelength.

### 2.3 Characterization of the Alumina Layer and Iron Catalyst Nanoparticles

The characteristics of the alumina and iron layers play a crucial role in determining the properties of the final CNT forest. As we have previously demonstrated in our work, the properties of the alumina layer significantly influenced the catalytic lifetime.^23^

To investigate the influence of the holder type on the evolution of the crystal structure and phase of 100 nm thick alumina layers on Si wafer after annealing at 700 and 900 °C, grazing incidence X-ray diffraction was performed using a Bruker D8 Discover. The measurements were conducted at a fixed incident angle of 5° with a 2θ range from 15° to 58°. Additionally, the mechanical properties of the alumina films were assessed using nanoindentation with a Hysitron TI 950 TriboIndenter. 10 nm deep indents were created with a Berkovich indenter at a loading rate of 1 nm/s, enabling the investigation of the film’s response to mechanical loading. A Horiba Jobin Yvon UVISEL instrument equipped with DeltaPsi2 software was used to perform spectroscopic phase modulated ellipsometry. The refractive index of the films was obtained and served as an indication of their density. Additionally, the surface morphology of these films and the catalyst nanoparticles formed by solid-state dewetting of a 1 nm-thick iron catalyst film were studied by using atomic force microscopy (AFM) with a Veeco Dimension 3100 V instrument.

## 3 Results and Discussion

### 3.1 *T*_c_ Affects the Macroscale Uniformity of CNT Forests

We investigated the impact of substrate holder design on the macroscopic uniformity of CNT forests grown under various *T*_c_ and *T*_g_ conditions. Notably, the results in Figure 1d–g demonstrate that regardless of the catalyst type, forests grown on Holder2 exhibit a dome-shaped nonuniform geometry with limited or poor growth at the edges. Figure S1 provides a detailed comparison of the spatial uniformity between forests grown using Holder1 and Holder2, with various *T*_c_ values employed during the catalyst nanoparticle formation step and a constant *T*_g_ of 700 °C during growth. While macroscopically uniform forests are grown at all *T*_c_ values with Holder1 (Figure S1a–j), the uniformity of forests grown on Holder2 depends on *T*_c_ (Figure S1k–t). We have shown that under growth conditions of *T*_c_ = 700 °C and *T*_g_ = 700 °C, both holder types can yield geometrically uniform CNT forests. However, it was observed that Holder2 improved the uniformity of CNT height across the forest and reduced the run-to-run variation in height and density of the forests.^26^ Therefore, it becomes crucial to reveal the underlying factors that determine the geometric uniformity of the forests when different holders are utilized. Such an understanding is essential to achieve consistent synthesis of uniform forests on large scales.

As mentioned earlier, the dynamic recipes offer a unique approach toward controlling the geometric uniformity of the CNT forests by tuning *T*_c_ and *T*_g_. The results presented in Figure 2 reveal the effect of varying *T*_c_ while maintaining a constant *T*_g_ of 700 °C on the uniformity of the as-grown forests. It is observed that as *T*_c_ increases from 400 to 1000 °C, the forest geometry undergoes a transition from nonuniform with a central hole at lower *T*_c_ values (400–600 °C) to uniform at moderate *T*_c_ values (700 and 800 °C) and back to nonuniform with a central hump at higher *T*_c_ values (900 and 1000 °C). Notably, all these experiments were conducted with the preheater temperature consistently set at 825 °C.

**Figure 2:**
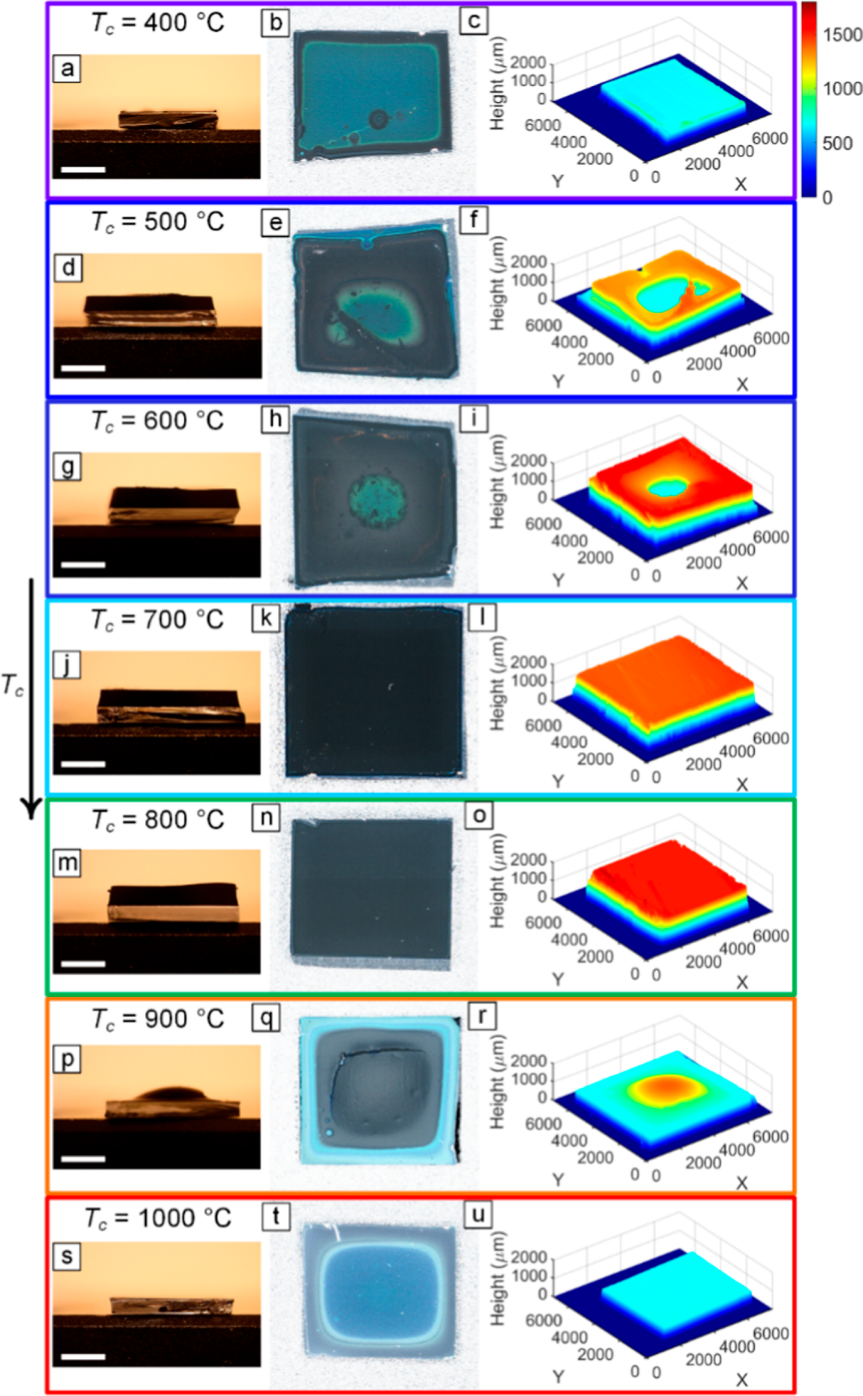
Effect of *T*_c_ on the uniformity of forests grown at *T*_g_ = 700 °C using Holder2 and Catalyst1. (a, d, g, j, m, p, s) Sidewall OM images, (b, e, h, k, n, q, t) top view images, and (c, f, i, l, o, r, u) 3D profilometry measurements provide various perspectives on the forest geometry. Preheater temperature was maintained at 825 °C for all cases. Scale bars in (a, d, g, j, m, p, s) show 2 mm.

Additionally, Figure S2 shows a similar trend in forest geometric uniformity when the preheater is not in operation (no preheating of the reactant gases prior to entering the reaction zone in which the catalyst chip is placed). We see similar trends, although the growth kinetics and final heights are affected by the preheater temperature, as shown in our previous work about decoupling gas-phase reactions.^22^ Importantly, the presence of the same trend with respect to the spatial distribution of growth across the macroscopic dimensions of the chip suggests that geometric uniformity is predominantly determined by the catalyst surface reactions rather than the gas phase reactions. These findings highlight the critical role of temperature distribution on the catalyst surface in governing spatial variation in the synthesis process, leading to nonuniform forest geometry.

For the case of a low *T*_c_ of 500 °C, SEM images in Figure 3a–g reveal the growth of a tangled CNT mat in the central region, transitioning abruptly to a vertically aligned CNT forest toward the edges. The presence of a distinct interface between these two morphologies confirms the collective growth model, proposed by Bedewy et al.^27,28^ This model stipulates the presence of a threshold density for the self-supporting structure of an aligned forest, where the crowding forces are sufficient for the liftoff of the growing forest. In the transition region, sidewall SEM images near the top (Figure 3c,d) demonstrate a high degree of CNT alignment, while toward the bottom of the forest (corresponding to the end stage of the growth), slightly lower alignment is observed (Figure 3f).

**Figure 3:**
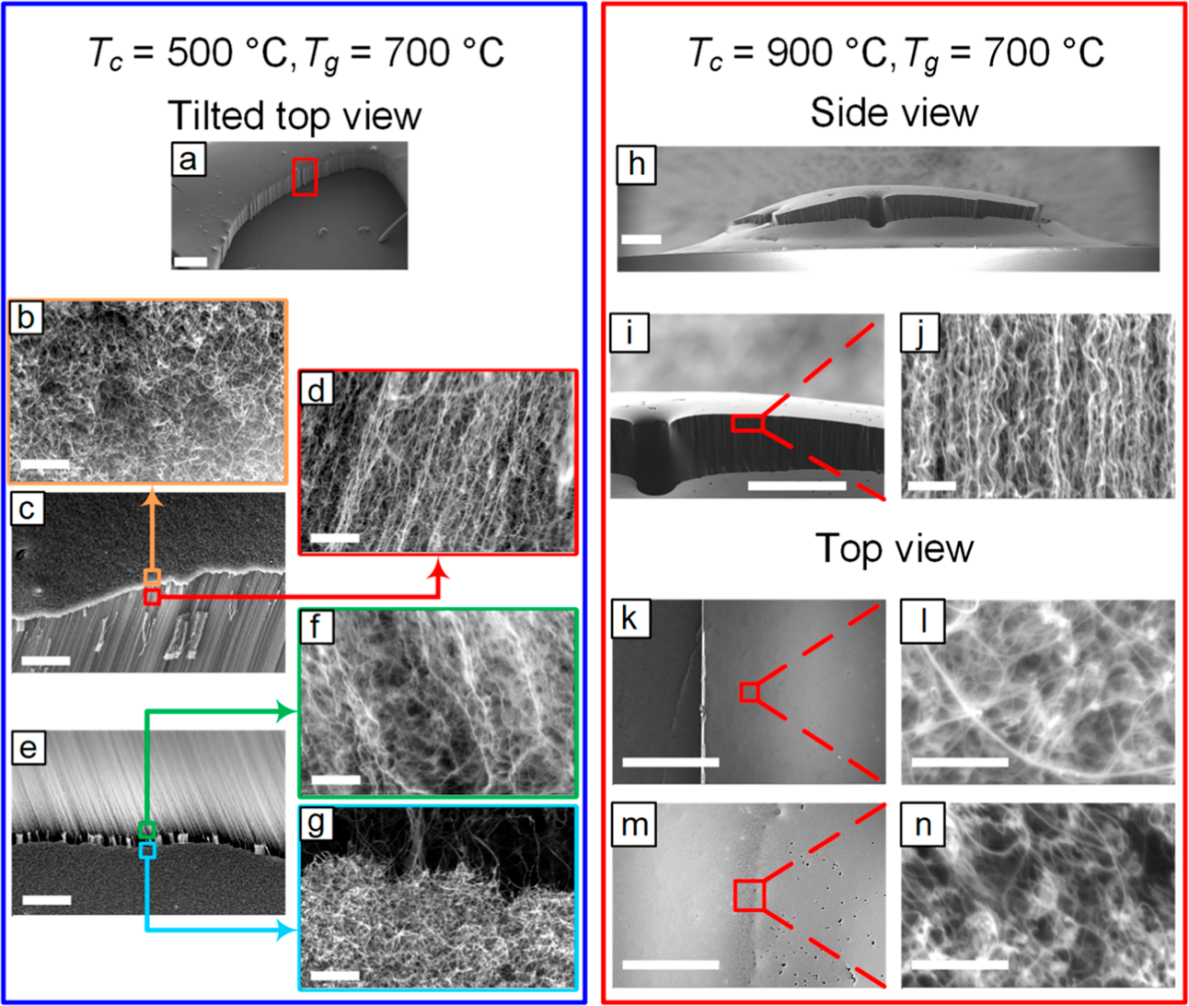
SEM images illustrate the spatial uniformity of the CNT forests grown after annealing at different temperatures. (a–g) At *T*_c_ = 500 °C, images show an abrupt transition from entangled CNT mats in the center to vertically aligned CNTs toward the edges of the sample. (h–n) At *T*_c_ = 900 °C, side-view SEM images demonstrate aligned CNTs grown in the center, while top view images show CNT growth across the chip, with the CNTs on the edges exhibiting lower alignment. Scale bars: (a, h, i, k, m) 400 μm, (b, d, f, g, j, l, n) 800 nm, and (c, e) 20 μm.

In contrast, for the high *T*_c_ of 900 °C, side-view SEM images in Figure 3h–j show the growth of a vertically aligned forest that increases in height toward the center of the sample. Top-view SEM images in Figure 3k–n show the growth of CNTs across the catalyst chip. Additional SEM images of this sample are provided in Figure S3. Comparing the side-view images in Figures 3j and S3b, we once again observe a higher degree of alignment near the top of the forest. Furthermore, comparing the top-view SEM images near the edge (Figure S3d) to the center of the forest (Figure S3f) reveals an increased CNT population density as we move toward the center. These observations once again suggest the presence of a density threshold for the growth of vertically aligned CNT forests. The cracking observed near the center, where CNTs grow taller, is known to result from the buildup of internal stresses due to mechanical and chemical coupling between neighboring individual CNTs with different growth rates.^29–32^

We used AFM to study the effect of the catalyst formation temperature (*T*_c_) on the distribution of the height of catalyst nanoparticles at both the center and the edge of catalyst-coated substrates. These samples underwent the catalyst formation step at various *T*_c_ values without proceeding to the subsequent CNT growth step. Figure S4 presents AFM images and the corresponding plots of nanoparticle height distribution. We observed that the samples treated at higher *T*_c_ values formed catalyst nanoparticles with increased heights and broader height distributions, in agreement with the concept of more rapid Ostwald ripening at elevated temperatures.^33^ These results agree with our previous work,^22^ as well as with findings in literature.^18,34^ Upon comparing the AFM images and height distribution plots of the center and edge regions of the catalyst at each *T*_c_ in Figure S4, we noted only a slight difference in nanoparticle height distribution between these areas across all cases, which cannot account for the observed geometric nonuniformity in CNT forest growth. This similarity of nanoparticle size distributions across the substrate at the beginning of the growth step suggests that the *T*_c_-dependent geometric nonuniformity stems from the chemical composition and phase of the nanoparticles rather than their size.

To further analyze the evolution of catalyst nanoparticle heights during growth, we conducted a mock growth experiment on a sample that underwent annealing at *T*_c_ = 500 °C. This sample was subjected to a growth temperature of *T*_g_ = 700 °C for 30 min without the introduction of hydrocarbon gases. Figure S5 shows that the height distribution of nanoparticles exhibited negligible variation between the center and the edge regions of the catalyst chip, once again suggesting that particle size does not explain the observed macroscopic nonuniformities.

Additionally, the areal number density of catalyst nanoparticles, calculated from AFM images using the watershed algorithm in Gwyddion software, remained relatively consistent between the edge and center regions of each catalyst chip. The corresponding values can be found in Table S1. In a previous study, we established a correlation between the density of CNTs and the activation percentage of catalyst nanoparticles, which is influenced by *T*_g_.^22^ Therefore, the spatial variations in the density of CNTs across a catalyst chip, observed in the SEM images (Figures 3 and S3), indicate a spatially varying activation percentage of catalyst nanoparticles across the catalyst-covered area. This observation supports our hypothesis that the temperature profile across the substrate accounts for CNT forest nonuniformity.

### 3.2 *T*_c_ Dependence of the Suitable *T*_g_ Range for CNT Growth

Figure 4a shows a typical growth recipe in which *T*_c_ and *T*_g_ are decoupled. Our CVD reactor’s capability in programming dynamic decoupled recipes has allowed us to investigate the independent effects of *T*_c_ and *T*_g_ on various aspects of forest growth, such as height^23^ and density.^22^ Our results reveal that at any constant *T*_c_, achieving geometrically uniform forest growth requires a specific *T*_g_ range. As shown by comparing the results in Figures 4 and 5, this *T*_g_ range for achieving uniform forest growth exhibits a shift toward higher values as *T*_c_ increases. For instance, when *T*_g_ is maintained at 700 °C, increasing *T*_c_ from 500 to 600 °C leads to a transition from a nonuniform to a uniform forest geometry. This relationship is schematically depicted in Figure 5m. These results demonstrate that at any constant *T*_c_, there exists a temperature range, within which CNT growth can occur, and this range shifts to higher temperatures with increasing *T*_c_.

**Figure 4:**
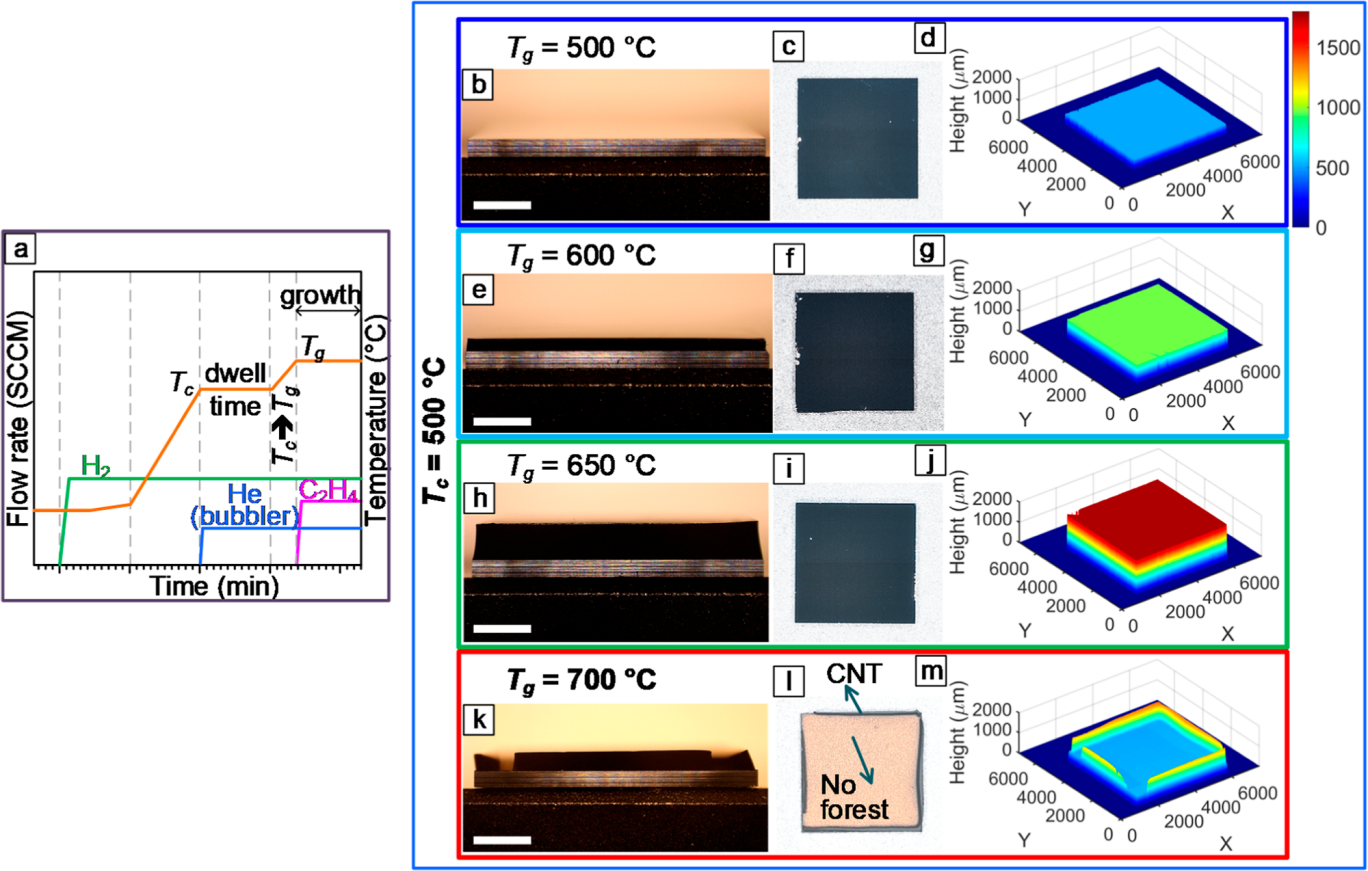
(a) A dynamic recipe showing the temperature profile of the substrate and the flow rate of gases. The effect of *T*_g_ on the spatial uniformity of CNT forests grown from Catalyst2 with *T*_c_ = 500 °C was evaluated by (b,e,h,k) sidewall OM images, (c,f,i,l) top view imaging of the forests, and (d,g,j,m) 3D profilometry. Scale bars in (b,e,h,k) are 2 mm.

**Figure 5:**
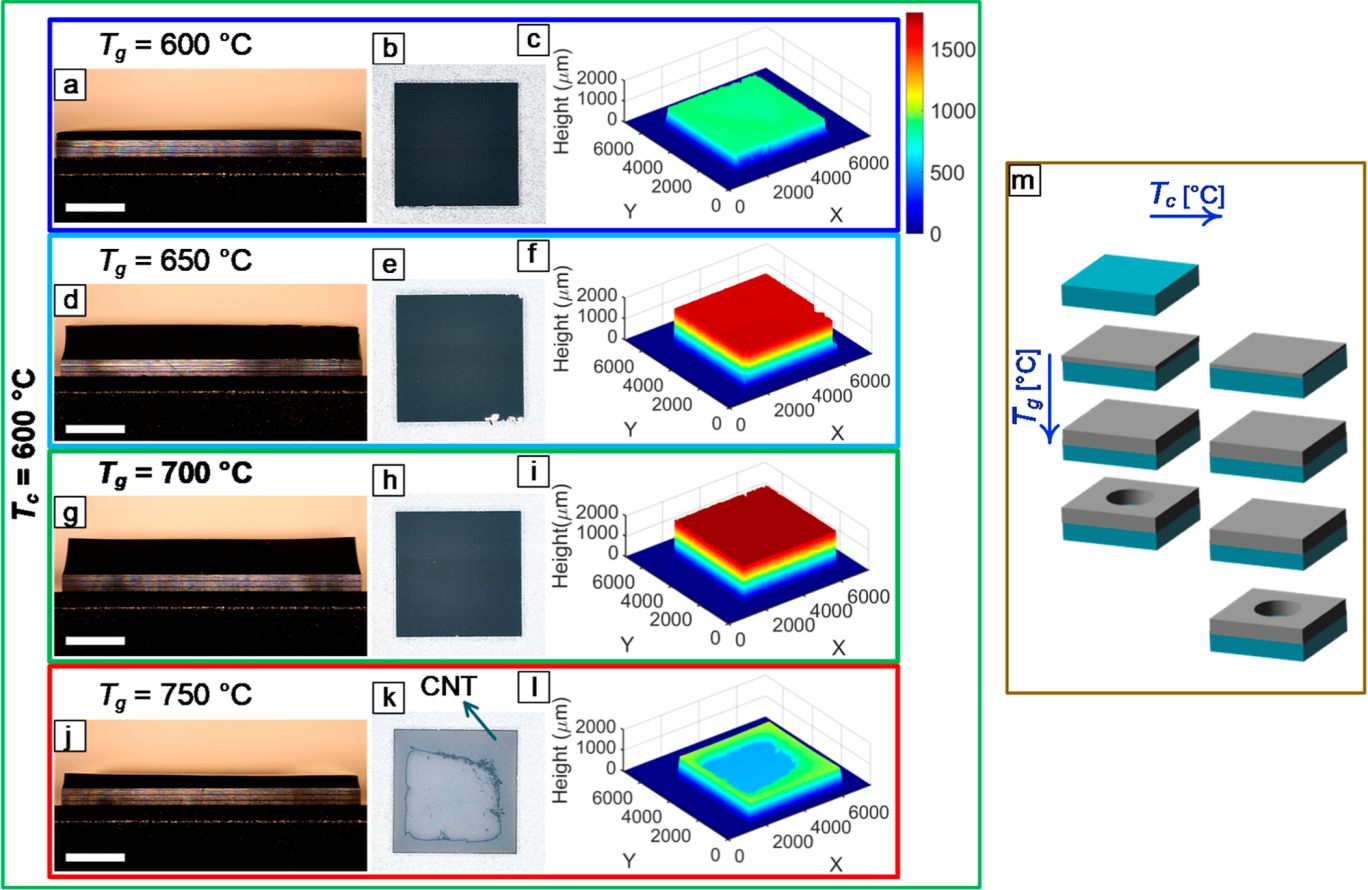
Effect of *T*_g_ on the spatial uniformity of CNT forests grown from Catalyst2 with *T*_c_ = 600 °C was evaluated by (a,d,g,j) sidewall OM images, (b,e,h,k) top view imaging of the forests, and (c,f,i,l) 3D profilometry. (m) The *T*_g_ range for achieving uniform CNT forests increases with increasing *T*_c_. Scale bars in (a,d,g,j) are 2 mm.

It is worth noting that at *T*_c_ = 600 °C and *T*_g_ = 700 °C, the growth was found to be nonuniform with Catalyst1 (Figure 2g–i), whereas the use of Catalyst2 resulted in a uniform forest growth (Figure 5g–i). This difference is attributed to the influence of catalyst thickness, stoichiometry, and other attributes on the growth behavior. Importantly, this variation in growth behavior is not solely attributed to the change in catalyst type but is indicative of the proximity of *T*_c_ = 600 °C to a critical threshold temperature for a transition to uniform growth. Hence, understanding the bounds of the growth conditions that are conducive to uniform growth is key for reducing run-to-run variations when operating at more forgiving process parameters. This insight into temperature bounds is key to understanding the factors governing CNT forest uniformity.

The observed changes in the *T*_g_ range or bounds for CNT forest growth as a function of *T*_c_ can be attributed to several contributing factors. First, it is known from the Gibbs–Thomson model that smaller nanoparticles exhibit lower melting temperatures than larger particles or bulk materials.^35^ In the context of CNT growth, this implies enhanced carbon diffusion into and on iron nanoparticles, leading to a further reduction in melting temperature and thus increased catalytic activity.^36^ Since we observe larger catalyst nanoparticles are formed at higher *T*_c_ (Figure S4), it follows that CNT growth from these larger nanoparticles formed at higher *T*_c_ would require a higher *T*_g_, following the Gibbs–Thomson model.

Second, researchers have investigated the impact of modifying the chemistry and structure of the catalyst support layer on catalytic activity through various methods, such as altering the deposition technique or utilizing ion beam bombardment.^37–39^ These studies have highlighted that catalytic activity strongly depends on factors such as porosity as well as the level of disorder, nonstoichiometry, and hydroxyl groups on the surface of oxide support. Consistent with these findings, we have also previously shown that densifying the alumina support layer via increasing the temperature in our rapid thermal pretreatment step significantly enhances the catalytic lifetime.^23^ Therefore, our current findings regarding the need for a higher *T*_g_ in samples that are subjected to higher annealing temperatures (*T*_c_) can be attributed to the formation of a denser, more ordered, and inert alumina support during the annealing step.

While the two factors mentioned above adequately explain the increase in the required *T*_g_ range for CNT forest growth with an increase in *T*_c_, they alone cannot account for the observed *T*_c_-dependent spatial nonuniformities of CNT forests. The existence of a temperature gradient across the catalyst chip causing these geometric nonuniformities combined with the above-mentioned temperature dependence is necessary to explain the trends shown in Figures 2, 4, and 5. We hypothesize that the design of the catalyst holder affects this temperature gradient and thus affects the geometric uniformity of the forest. While this temperature gradient across the catalyst chip might not be significant enough to induce a gradient in nanoparticles’ height during annealing and growth, it could alter the phase and oxidation state of nanoparticles across the chip. For instance, He et al. have shown that the phase of iron catalyst (α-Fe or Fe_3_C) depends on the growth temperature (with only Fe_3_C observed below 600 °C and both phases present at higher temperatures), and this phase difference significantly affects the growth rate of CNTs.^40^ In our case, it appears that the temperature gradient across the chip has a significant effect on catalyst activation and growth after the introduction of hydrocarbon gases, resulting in the observed geometric nonuniformities and edge effects.

To compare the kinetics and apparent activation energies of CNT growth versus ripening of catalyst nanoparticles, we analyzed the growth kinetics of CNT forests grown at various *T*_c_ and *T*_g_. Real-time videography and image processing techniques, as described in our previous work,^41^ were employed for this purpose. The growth kinetics curves are presented in Figure S6. The initial growth rate was determined for each growth condition, showing an increase at higher *T*_g_ values at each *T*_c_. This observation is consistent with the Arrhenius nature of the CNT growth. To determine the apparent activation energy (*E*_a_), we plotted the natural logarithm of the growth rates against 1/*T*_g_ [*K* ^–1^] at each *T*_c_ and derived the activation energy from the slope of the linear fits to these Arrhenius plots. The obtained activation energies were then plotted against *T*_c_, as shown in Figure S6i. We found that the activation energy values did not exhibit significant variation with *T*_c_. Moreover, these values are in line with those reported in the literature.^42–44^ Meshot et al.^44^ reported activation energy values in the range of 98–181 kJ/mol, with higher values corresponding to higher preheater temperatures (set at 980–1120 °C). Given that our preheating temperature was set at 825 °C, the slightly lower activation energies observed in our study are consistent with this difference in preheating temperature conditions.

It is insightful to compare CNT growth kinetics to the kinetics of catalyst nanoparticle ripening. Several studies in the literature have utilized in situ TEM observations and atomistic simulations to demonstrate that the ripening mechanism of small catalyst nanoparticles (2–5 nm) is predominantly governed by Ostwald ripening, rather than particle migration and coalescence.^45,46^ While larger particles (10 nm or larger) are more likely to undergo migration and coalescence when near each other, there are reports demonstrating that even with Ostwald ripening of immobile particles, certain particles can grow significantly larger. Researchers have proposed models for Ostwald ripening that can be either interface-controlled or diffusion-controlled. Given the low activation barrier for diffusion of typical metal adatoms on substrates, ripening kinetics is typically interface controlled.^46,47^ The apparent activation energy for ripening, *E*_tot_, can be calculated by *E*_tot_ = ΔH_sub_ – *E*_ads_ + *E*_diff_, where Δ*H*_sub_ represents the heat of sublimation of the metal, *E*_ads_ is the energy for metal adatom adsorption on the substrate, and *E*_diff_ is the energy for adatom diffusion on the substrate surface. Hence, *E*_tot_ depends on the specific metal and support, with reported values of 141 kJ/mol for Pb on MgO and 264.5 kJ/mol for Ni nanoparticles on MgAl_2_O_4_ substrate.^46,47^ It is noteworthy that Fe has a Δ*H*_sub_ close to Ni (416 vs 430 kJ/mol) and comparable typical values for metal adatom adsorption energy (*E*_ads_) and adatom diffusion energy (*E*_diff_) on alumina. Hence, we can infer that the *E*_tot_ for Fe nanoparticle sintering on alumina would likely fall within the range 250 kJ/mol.

In the context of comparing the kinetics of CNT growth with the kinetics of nanoparticle size evolution, both processes exhibit an exponential relationship with temperature. While the exact values of the constants in the kinetic equations are required to determine which rate is more sensitive to temperature gradients across the catalyst chip, the higher values of activation energy, as well as the presence of two exponential terms in the kinetics of sintering,^46,47^ suggest a potential for greater sensitivity to temperature variations compared to CNT growth kinetics. This higher dependence of catalyst nanoparticle ripening on temperature can account for spatial variations of deactivation behavior in the presence of spatial temperature gradients. However, this higher temperature dependence does not account for variations in early growth at the steps of catalytic activation and CNT liftoff because of the uniformity of nanoparticle size measurements shown in Figures S4 and S5. AFM measurements did not reveal significant changes in the nanoparticle size across the catalyst chip. Importantly, it is well-known that factors such as particle size, shape, and phase play crucial roles in the growth kinetics from each nanoparticle. Consequently, it can be inferred that the phase of particles is influenced by temperature gradients across the chip, contributing to the observed differences in growth kinetics from individual nanoparticles at different locations on the chip and contributing to the observed geometric nonuniformities.

### 3.3 Suggested Mechanism Based on a Temperature Profile across the Catalyst Chip

Based on the findings above, we hypothesize the presence of a steep temperature profile across the catalyst chip when utilizing Holder2, a 2 in. recessed Si holder. As schematically illustrated in Figure 6a, this temperature profile, in conjunction with the increasing *T*_g_ range for CNT forest growth at higher *T*_c_, explains the *T*_c_ dependence of geometric uniformity observed in this study. Importantly, while the temperature gradient across the catalyst chip may not be enough to induce nonuniformities in catalyst nanoparticle size distribution, it plays a pivotal role in catalyst activation, CNT growth rate, and catalyst deactivation, as discussed above. Next, we correlate the variations of *T*_c_ and *T*_g_ with CNT crystallinity.

**Figure 6:**
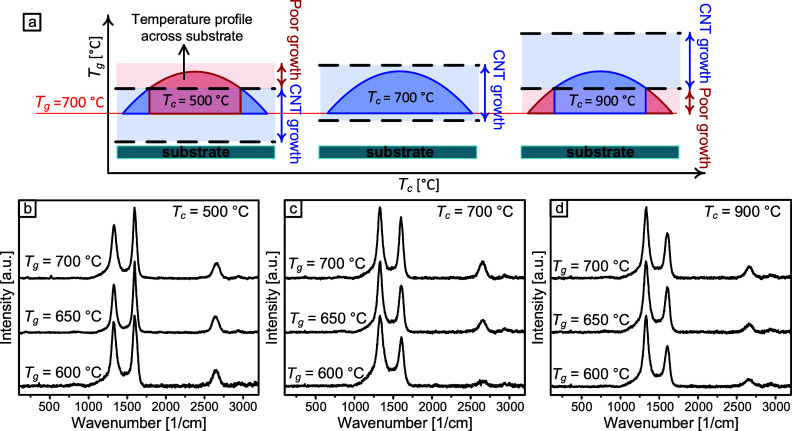
(a) A hypothesized temperature profile across the catalyst chip that explains the experimental observations on the effect of *T*_c_ on the geometric uniformity across the CNT forests. (b–d) Representative top-view Raman spectra from the CNT forests grown at various *T*_g_ values after annealing at (b) *T*_c_ = 500 °C, (c) 700 °C, and (d) 900 °C show the dependence of the structural quality on the growth conditions, suggesting that the growth of high-quality CNTs is achieved at a low *T*_c_ and a high *T*_g_.

Raman spectra from multiple points on the top surface of forests grown under different *T*_c_ and *T*_g_ conditions were obtained to assess the structural quality of the CNTs. Representative spectra are shown in Figure 6b, and the analysis of the *I*_G_/*I*_D_ ratios as a function of *T*_g_ for various *T*_c_ values is displayed in Figure S7a. Consistent with our findings, the results show an increase in the *I*_G_/*I*_D_ ratio with higher *T*_g_, which indicates improved structural quality due to the effect of higher *T*_g_ on reducing defects by thermal healing. It is worth mentioning that the data was collected from areas exhibiting vertically aligned forest. Additionally, as shown in Figure S7b–d, the analysis reveals a dependence of the *I*_G_/*I*_D_ ratio on *T*_c_ at a constant *T*_g_, although this dependence becomes weaker as *T*_g_ increases. This observation aligns with our previous finding of a negligible influence of *T*_c_ on the *I*_G_/*I*_D_ ratio at *T*_g_ = 720 °C.^23^ Overall, our results suggest that the highest *I*_G_/*I*_D_ values and thus the best structural quality are obtained at lower *T*_c_ and higher *T*_g_, as exemplified by the uniform forest grown at *T*_c_ = 500 °C and *T*_g_ = 650 °C (Figure 4h–j). Therefore, our approach allows for the optimization of CNT crystallinity while ensuring the forest macroscopic uniformity of geometry.

In addition to the comparisons shown in Figure S1, here, we provide further experimental proof for the effect of substrate holder design on the evolution of temperature across the catalyst chip. We performed a comparative study, in which we utilized Si chips with a 100 nm thick alumina layer as samples and annealed them on Holder2 at *T*_c_ = 700 and 900 °C. Various characterization techniques including XRD, ellipsometry, nanoindentation, and AFM were employed to analyze these samples. The characterization results were then compared with those from identical samples annealed on Holder1 using the same recipe. Notably, samples with a 10 nm thick alumina layer were used for AFM analysis. The XRD spectra presented in Figure 7a show that the as-deposited alumina layer is primarily amorphous. The peaks at 2Θ = 35.8° and 41.7° appear in all samples and are assigned to orthorhombic Al_2_SiO_5_ due to the formation of an AlO_*x*_–SiO_*y*_ interphase. Furthermore, the peak at 2Θ = 32° appears in the spectra of all annealed samples and corresponds to the formation of crystalline tetragonal γ phase of alumina. Additional peaks at 2Θ = 19.4° and 2Θ = 46.4° are observed in the spectra of the sample annealed at 900 °C on Holder1, as well as in the samples annealed at 700 and 900 °C on Holder2. These peaks indicate further crystallization of alumina into monoclinic Θ phase. Simultaneous to the formation of these two peaks, a weakening of the peak at 2Θ = 32° is observed in these samples. The resemblance of the peaks observed in the sample annealed on Holder2 at 700 °C to those seen in the samples annealed at 900 °C on both holders suggests that the sample on Holder2 experiences higher temperatures under similar process parameters for the annealing step. This finding provides compelling evidence of temperature variations across the chip, influenced by the choice of substrate holder.

**Figure 7:**
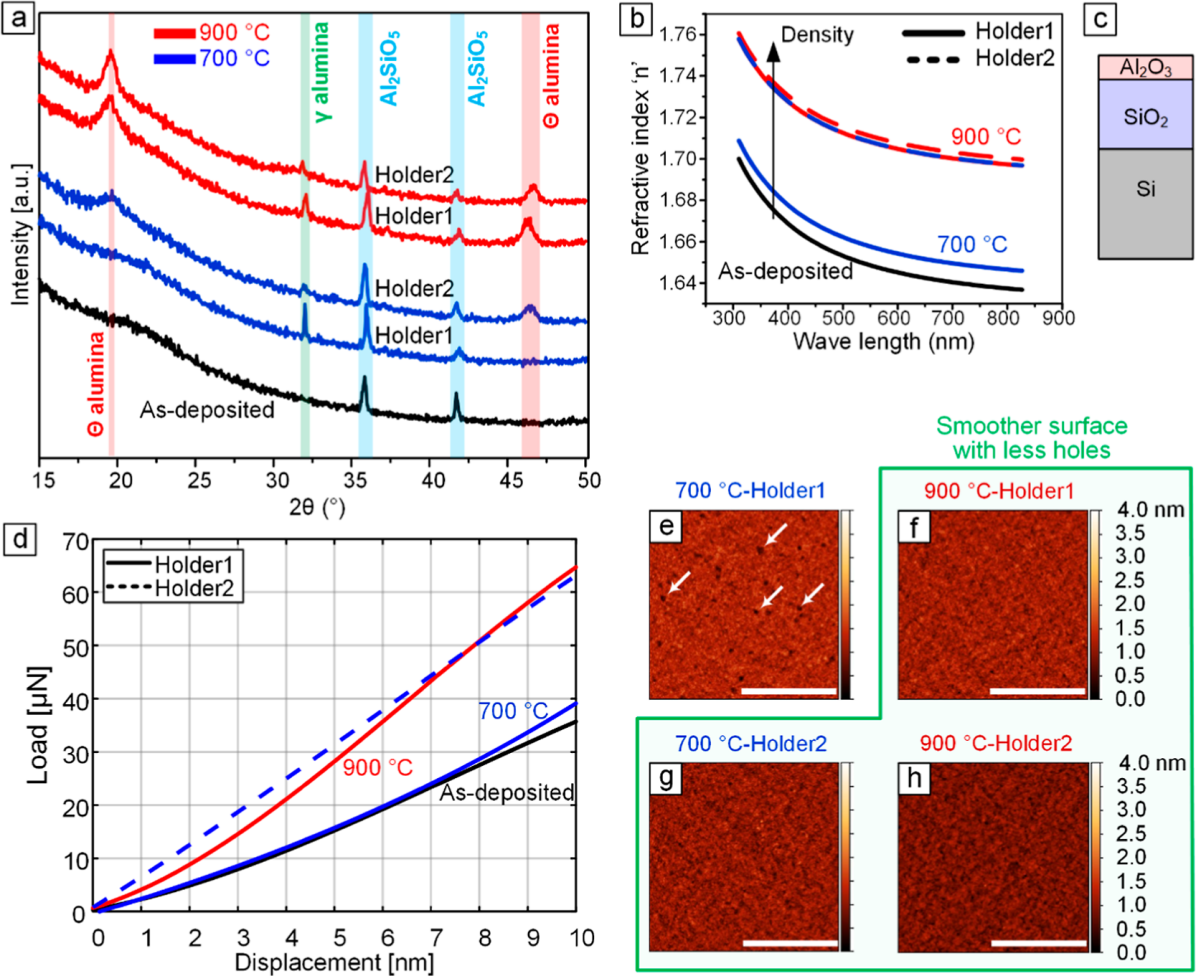
Characterization of alumina films annealed on two substrate holders at 700 and 900 °C. (a) XRD spectra reveals the emergence of crystalline peaks after annealing at 700 °C on Holder2, resembling the peaks observed after annealing at 900 °C on Holder1. (b) The refractive index, calculated as a measure of film density using the optical model in (c), shows that annealing at 700 °C on Holder2 densifies the film similar to annealing at 900 °C on Holder1. (d) The loading portion of nanoindentation confirms higher density of alumina after annealing at 700 °C on Holder2. AFM images of annealed alumina at (g) 700 °C and (h) 900 °C on Holder2 show a smoother surface, comparable to (f) the sample annealed at 900 °C on Holder1, with fewer holes than (e) the sample annealed at 700 °C on Holder1. Distinct holes are indicated by white arrows in (e). The scale bars in (e–h) represent 500 nm.

Ellipsometry was performed to study the effect of the annealing temperature and substrate holder type on the refractive index of alumina films using the optical model shown in Figure 7c (Al_2_O_3_/SiO_2_/Si) for data fitting. The Sellmeier dispersion function $n\left(\lambda \right)=\sqrt{A+\frac{B\lambda^{2}}{\lambda^{2}-\lambda_{1}^{2}}}$ with fixed parameters of *B* = 1.32 and λ_1_ = 121.59 nm was chosen for the alumina layer based on previous studies in the literature.^48,49^ The refractive index (*n*) as a function of wavelength is plotted in Figure 7b for various annealing conditions of the alumina layers. It can be inferred that the density of the alumina layer annealed at 700 °C on Holder2 is noticeably higher compared to that of the sample subjected to a similar annealing condition on Holder1.

The load–displacement curves obtained from nanoindentation tests on 100 nm-thick alumina films deposited on SiO_2_/Si substrates are depicted in Figure 7d. Notably, the load–displacement curve of the sample annealed at 700 °C on Holder2 exhibited behavior similar to that of the film annealed at 900 °C on Holder1. This finding is consistent with the results of other characterization techniques, indicating that Holder2 led to the formation of denser and more crystalline films compared to Holder1 under similar annealing conditions at *T*_c_ = 700 °C.

The AFM images of annealed alumina on Holder2 at 700 and 900 °C are depicted in Figure 7g,h, respectively. These images show a smoother surface, resembling the sample annealed at 900 °C on Holder1 (Figure 7f), and with fewer observable holes compared to the sample annealed at 700 °C on Holder1 (Figure 7e). The AFM image of the sample annealed at 700 °C on Holder1 shows distinct holes, as indicated by the white arrows in Figure 7e.

Taken together, the comprehensive characterization of the alumina films provides insight into the effects of the substrate holder type on the growth process. The observed higher temperatures experienced in the central region of the catalyst chip on Holder2, in comparison to holder1, indicate the existence of a temperature profile across the chip. Collectively, these findings provide compelling evidence that the choice of substrate holder type affects the temperature profile experienced by the catalyst chip, the properties of the alumina layer, and thus the CNT growth process.

### 3.4 *T*_c_ Dependence of Forest Geometric Uniformity Is Affected by Annealing Time

Catalyst nanoparticle formation through dewetting of the catalyst film during the annealing process is a critical factor in achieving reproducibility and scalability in the CNT growth process. Catalyst nanoparticle formation is influenced by several parameters, with the annealing time and temperature being the most critical factors with the greatest impact. The significance of annealing time and temperature is supported by the fact that the two primary deactivation mechanisms involved, namely, Ostwald ripening and subsurface diffusion, are both dependent on time and temperature. Pander et al.^50^ have demonstrated that the formation of catalyst particles during the annealing process depends on both time and temperature. Their research highlights the interplay between the rates of Ostwald ripening and subsurface diffusion at different annealing times and temperatures as the primary determining factor. Optimum catalyst pretreatment condition involves appropriate catalyst reduction to a metallic state and minimized Ostwald ripening.^51^ Carpena-Nunez et al.^52^ found a positive correlation between the extent of catalyst reduction and the density of CNT arrays, underscoring the importance of adequately reducing the catalyst for efficient CNT nucleation and growth. Research has demonstrated that maintaining a highly reductive environment during annealing, through exposure to trace amounts of carbon^53^ and the elimination of CO_2_,^54^ can aid in reducing Fe oxide and achieving a greater number density of active catalyst particles with a longer lifespan for subsequent CNT nucleation and growth processes.

In contrast to most studies on the effect of catalyst pretreatment time and temperature, which typically employ relatively long times greater than 3 min,^51,55^ our study demonstrates that a maximum annealing time of 1 min is sufficient. Results in Figure 8 show that the annealing time needed to obtain a uniform forest geometry depends on *T*_c_. Specifically, for lower values of *T*_c_ (700 °C), a longer annealing time of 25 s is necessary to achieve uniform forest growth, whereas for higher values of *T*_c_ (800 °C), uniform forest growth can be attained in a shorter annealing time. It is worth mentioning that in the case of decoupled recipes, the shortest time possible to go from a higher *T*_c_ to a lower *T*_g_ is 20 s due to the existing thermal mass. During this time, the catalyst nanoparticles keep evolving, and thus, the lowest possible time to study the effect of annealing time at *T*_c_ = 800 °C is 20 s, which results in a geometrically uniform forest. This *T*_c_ dependence is possibly due to the difference in kinetics of reduction of catalyst nanoparticles at different *T*_c_ values, i.e., faster reduction kinetics at higher *T*_c_. As mentioned earlier, it is known that reduced iron is the active phase of catalyst nanoparticles for growing CNT.^52–54,56^ Figure 8 shows that the uniformity of the forests improves by increasing the annealing time from 15 to 25 s at *T*_c_ = 700 °C, highlighting the utility of optimizing the annealing time at constant temperature for maximizing the geometric uniformity of CNT forests.

**Figure 8:**
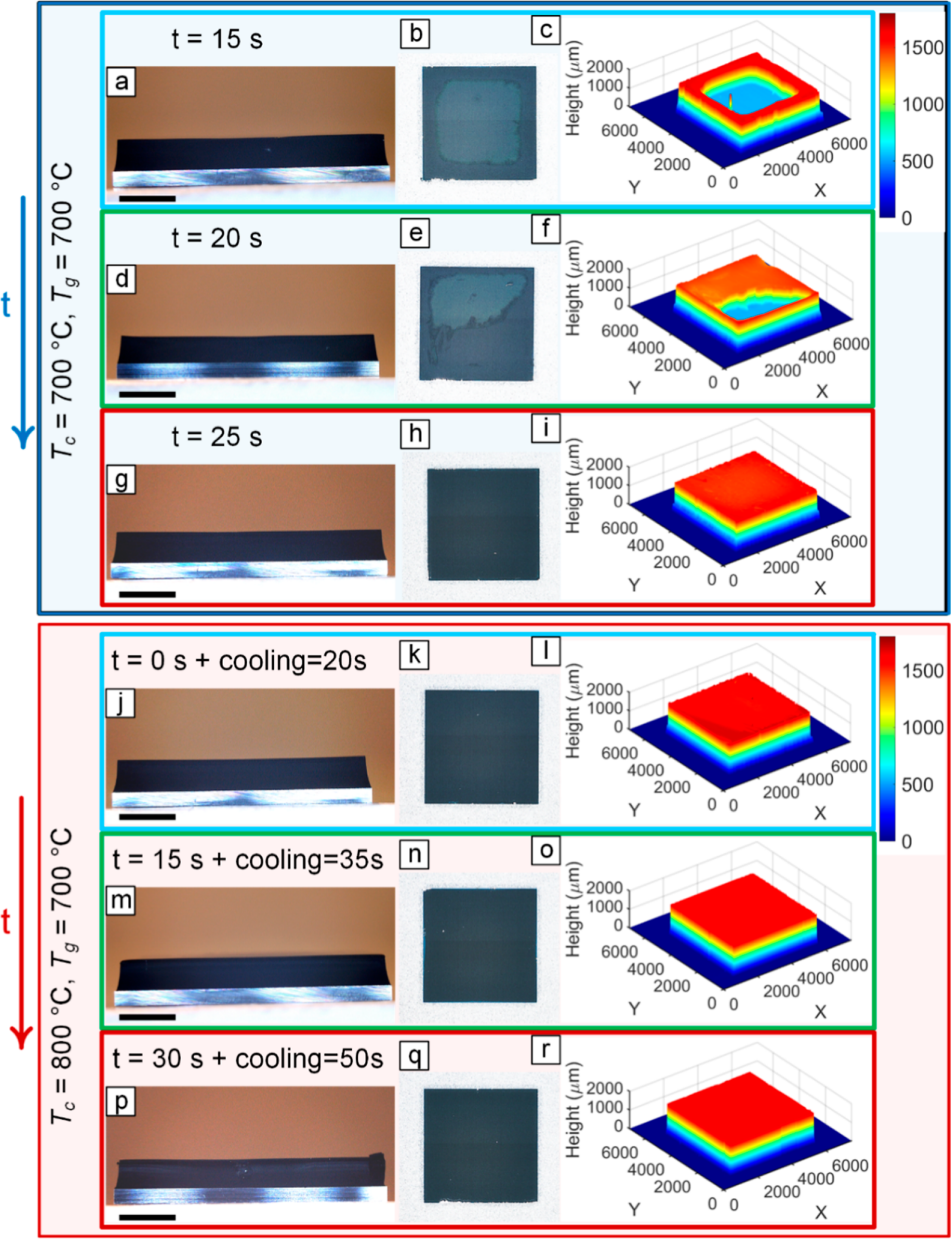
Effect of annealing time at *T*_c_ = 700 and 800 °C on uniformity of CNT forests grown at *T*_g_ = 700 °C with Holder2. (a,d,g,j,m, p) Sidewall OM images with scale bars showing 2 mm, (b,e,h,k,n,q) top view images of the forests, and (c,f,i,l,o,r) 3D profilometry shows that longer annealing is required to grow geometrically uniform forests at a lower *T*_c_ of 700 °C, while uniformity is achieved with shorter annealing time at a higher *T*_c_ of 800 °C.

## 4 Conclusion

In this study, we investigated the effect of substrate holder type and dynamic recipe parameters on the geometric uniformity of VACNT forests grown using a custom-designed RTP-CVD reactor. We demonstrate that the type of substrate holder affects the geometric uniformity in addition to reducing variability. Additionally, dynamic recipes enable unique control over the geometric uniformity of the CNT forests. We found that the catalyst treatment temperature, *T*_c_, affects the shape of the nonuniformities, with low *T*_c_ values resulting in empty regions in the center of the catalyst chip and high *T*_c_ values resulting in a hump at the middle with taller CNTs. This trend is attributed to the interplay between spatial temperature distribution across the chip and surface reactions rather than gas phase reactions. Our AFM analysis of the evolution of catalyst nanoparticle size during annealing and during mock growth shows that variations in nanoparticle sizes cannot account for the observed geometric nonuniformity in the forest. We show that a limited range of growth temperature, *T*_g_, is suitable for uniform CNT forest growth at each *T*_c_. This *T*_g_ range increases with increasing *T*_c_. Based on Raman spectroscopy, we show that the optimal quality and crystallinity of CNTs are achieved at low *T*_c_ (500 °C) and high *T*_g_. Hence, tuning *T*_g_ values to obtain a uniform forest at low *T*_c_ and the highest possible *T*_g_ is the optimal condition. In sum, our study sheds light on the impact of dynamic recipe parameters on CNT forest uniformity and reveals how substrate holder design can influence the geometric uniformity of forests grown in infrared-heated cold-walled reactors. We provide evidence for our hypothesis that the temperature profile across the substrate underlies the observed geometric nonuniformities. Hence, characterizing and modeling the spatiotemporal evolution of temperature across the catalyst-coated chips is crucial for reactor design to either achieve large-scale uniformity or tailored profiles for desired 3D forest geometries. This understanding is crucial for the robust manufacturing of CNT forests for various applications such as sensors, electronics, and membranes.

## Supplementary Material



## Notes

Effect of substrate holder design on the geometric uniformity of CNT forests grown with various dynamic recipes; effect of *T*_c_ on the uniformity of CNT forests grown at *T*_g_ = 700 °C when preheater was not used; AFM images of the edge and center of the catalysts annealed at various *T*_c_ to study the height distribution of catalyst nanoparticles formed during solid-state dewetting at various *T*_c_; AFM images of the edge and center of the catalyst annealed at *T*_c_ = 500 °C and subsequent mock growth at *T*_g_ = 700 °C for 30 min to analyze the evolution of catalyst nanoparticles; kinetics plots at various *T*_c_ and *T*_g_ values and corresponding activation energies obtained from Arrhenius plots; and *I*_G_/*I*_D_ ratios at various combinations of *T*_c_ and *T*_g_ to compare the structural quality of CNTs grown at each condition (PDF)
