# Supplementary material for: Improving Geometric Uniformity in Dynamic Chemical Vapor Deposition of Carbon Nanotube Forests
Source: Ind Eng Chem Res. 2025 May 27;64(23):11327–39. doi: 10.1021/acs.iecr.4c03787 (PMC12164265; doi:10.1021/acs.iecr.4c03787)
Supplement: Supplementary file 1 [file ie4c03787_si_001.pdf]

Supplementary Information for

Improving Geometric Uniformity in Dynamic Chemical Vapor Deposition of Carbon Nanotube Forests

Golnaz Najaf Tomaraei[1,2], Moataz Abdulhafez[2], Soumalya Ghosh[1,2], Jaegeun Lee[2,3], and Mostafa Bedewy[1,2,4]*

[1]Department of Mechanical Engineering and Materials Science, University of Pittsburgh, 3700 O'Hara Street, Pittsburgh, PA 15261, USA

[2]Department of Industrial Engineering, University of Pittsburgh, 3700 O'Hara Street, Pittsburgh, PA 15261, USA

[3]School of Chemical Engineering, Pusan National University, 2, Busandaehak-ro 63 beon-gil, Geumjeong-gu, Busan, 46241, Republic of Korea

[4]Department of Chemical and Petroleum Engineering, University of Pittsburgh, 3700 O'Hara Street, Pittsburgh, PA 15261, USA

*Author to whom correspondence should be addressed.

E-mail address: mbedewy@pitt.edu (M. Bedewy)

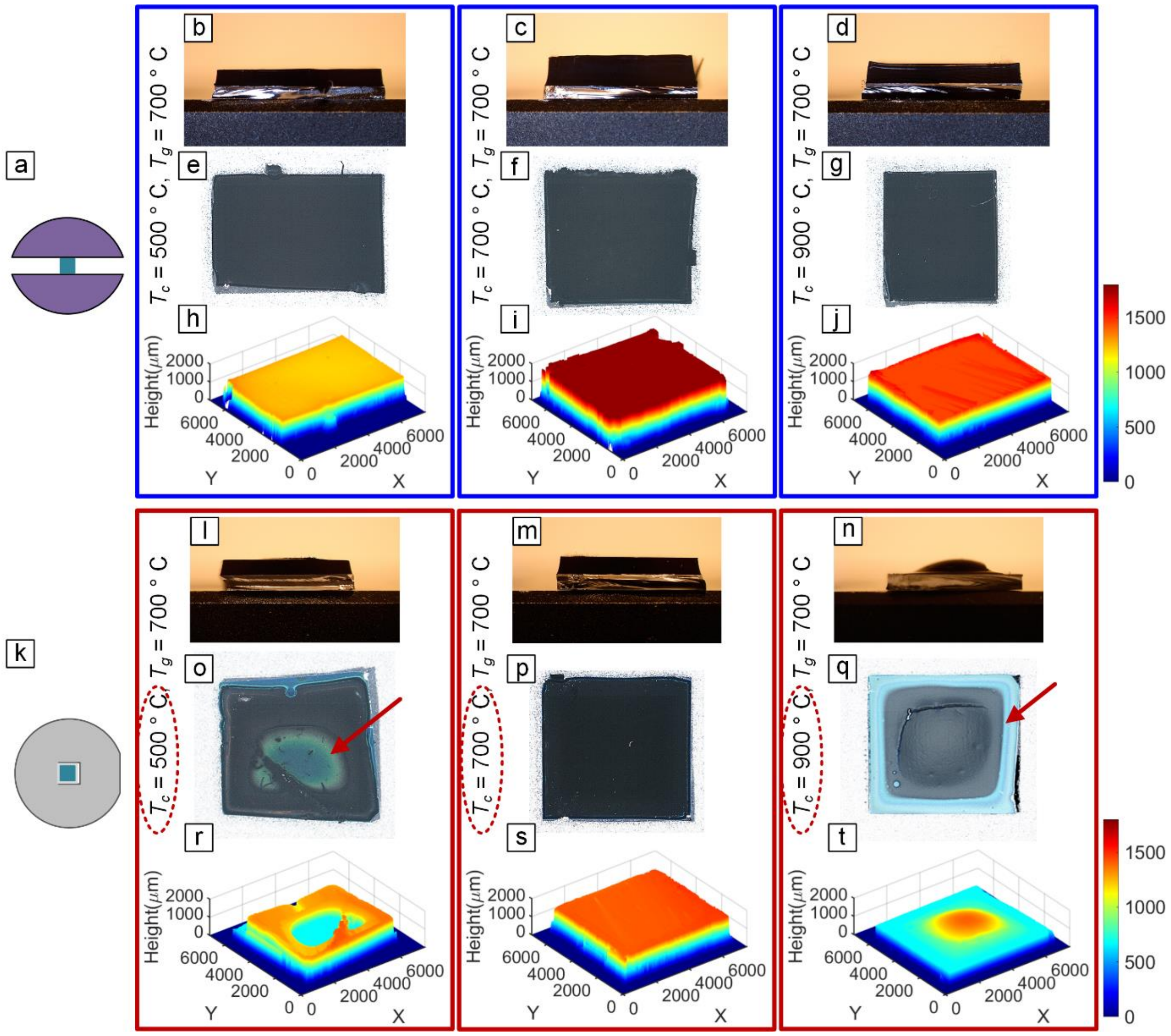

Fig. S1. Schematics illustrating the two types of substrate holders used in this study: (a) Holder1 and (k) Holder2. CNT forests grown on Holder1 at various T_c with a constant T_g of 700 °C exhibit uniform geometry, as demonstrated by (b-d) side-view images, (e-g) top-view images, and (h-j) 3D profiles. In contrast, the spatial uniformity of CNT forests grown on Holder2 at a constant T_g of 700 °C depends on Tc, as depicted by (l-n) side-view images, (o-q) top-view images, and (r-t) 3D profiles.

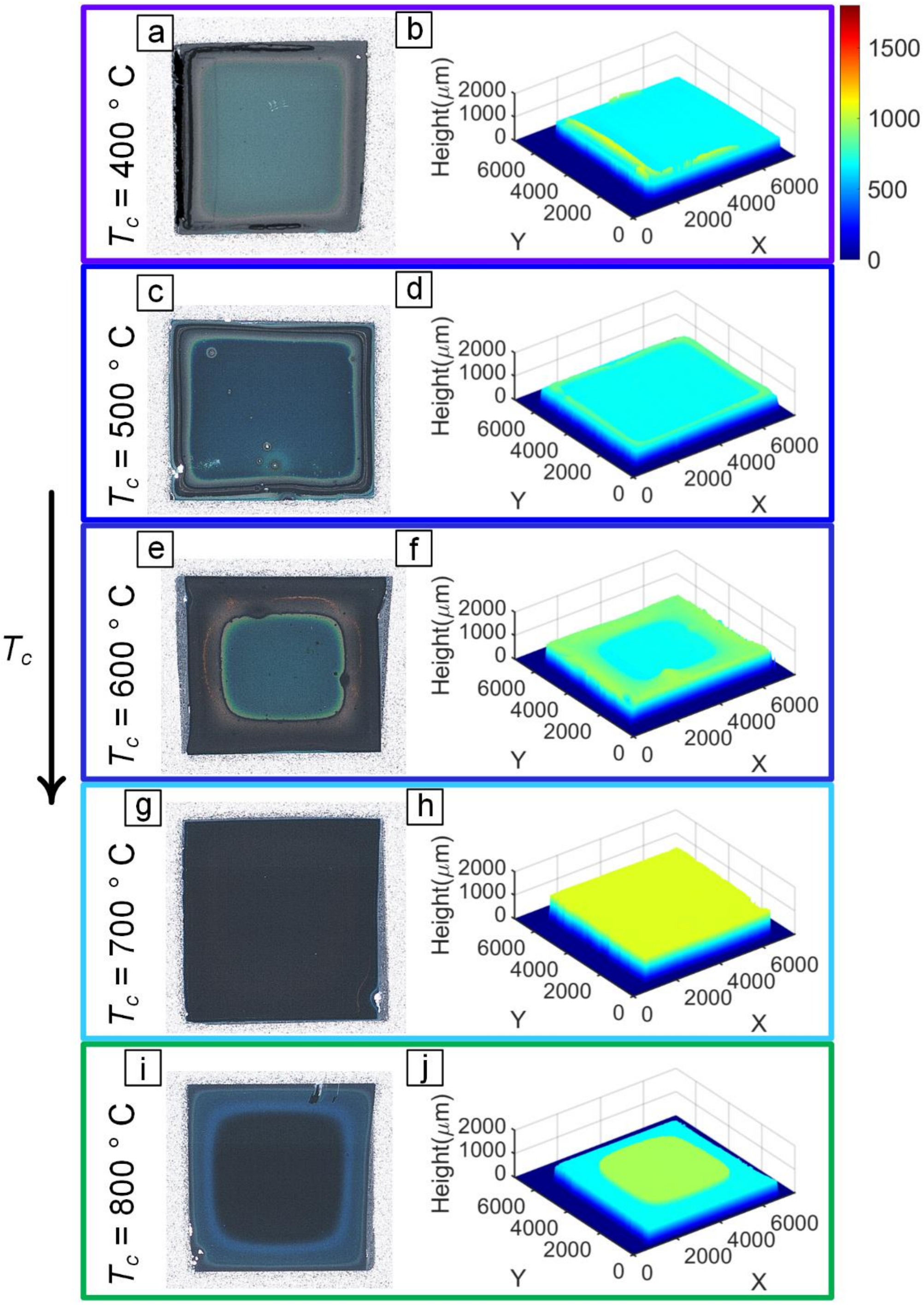

Fig. S2. Effect of T_c on the uniformity of CNT forests grown using holder2 at T_g = 700 °C. (a, c, e, g, i, k, m) top view images of the forests and (b, d, f, h, j, l, n) 3D profilometry measurements provide details of the forest geometry. Preheater was off in all cases.

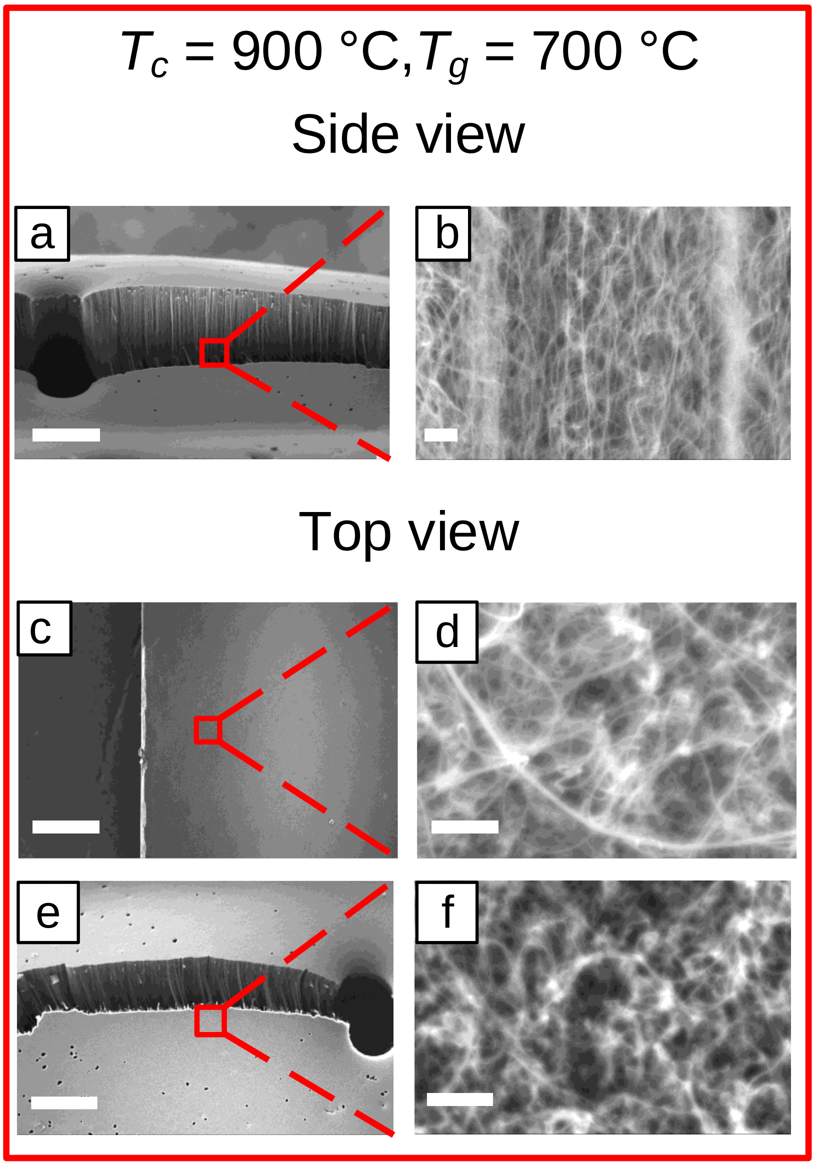

Fig. S3. Additional SEM images of the CNT forest grown after annealing at 900 °C. (a) Low magnification side-view image and (b) high magnification side-view image reveal good alignment of CNTs closer to the bottom, representing the later stages of growth. (c) Low magnification top-view image near the edge and (d) high magnification top-view image near the edge highlight the density and alignment of CNTs. (e) Low magnification top-view image near the center and (f) high magnification top-view image near the center show higher density and alignment of CNTs near the center of the sample. Scale bars: (a, c, e) 200 µm and (b, d, f,) 400 nm.

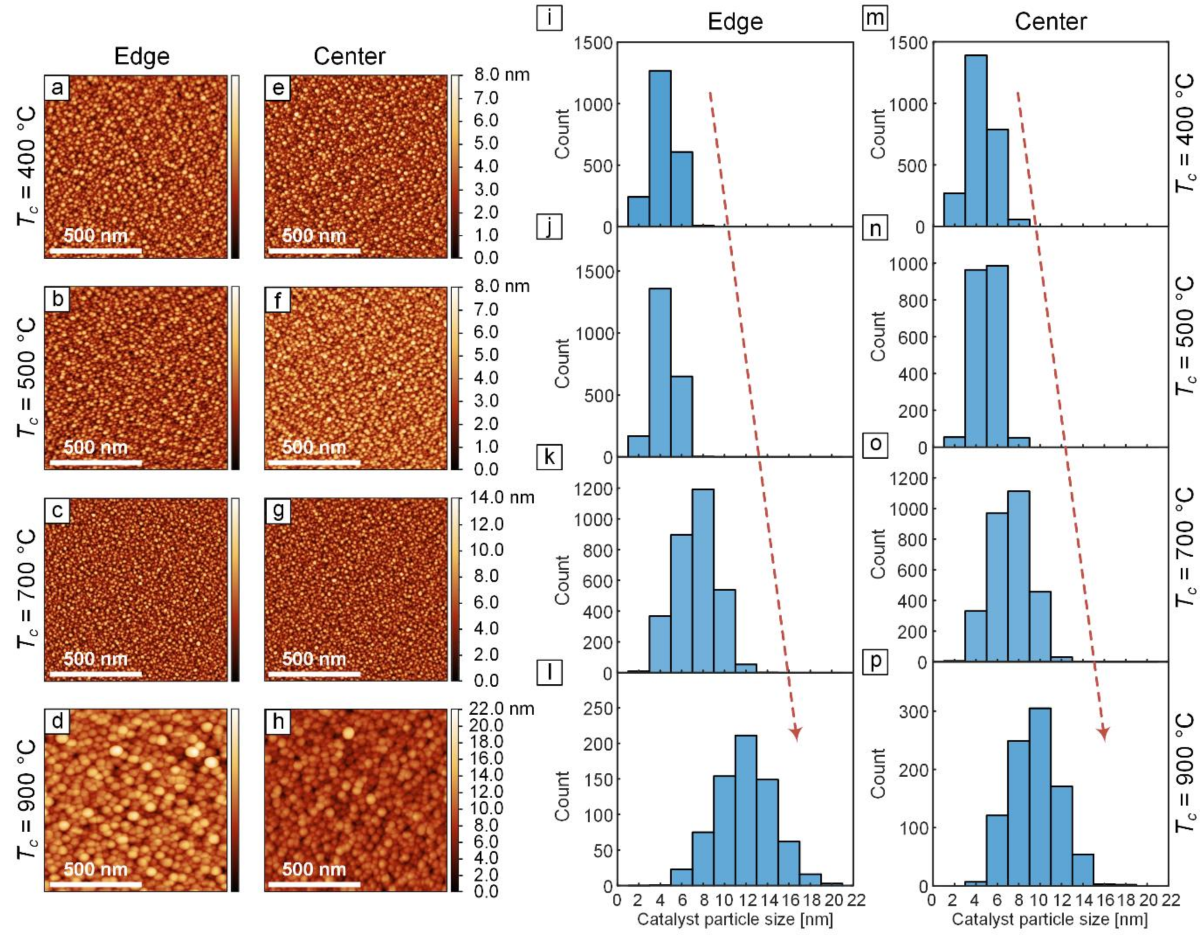

Fig. S4. Top view AFM images of (a-d) the edge and (e-h) the center of the catalyst chips annealed at various T_c. The corresponding heigh distribution of catalyst nanoparticles plotted as bar charts in (i-p) show that the height of catalyst nanoparticles increase with Tc at both the edge and the center of catalyst chips, while the difference in height of nanoparticles between the edge and center of each sample is insignificant.

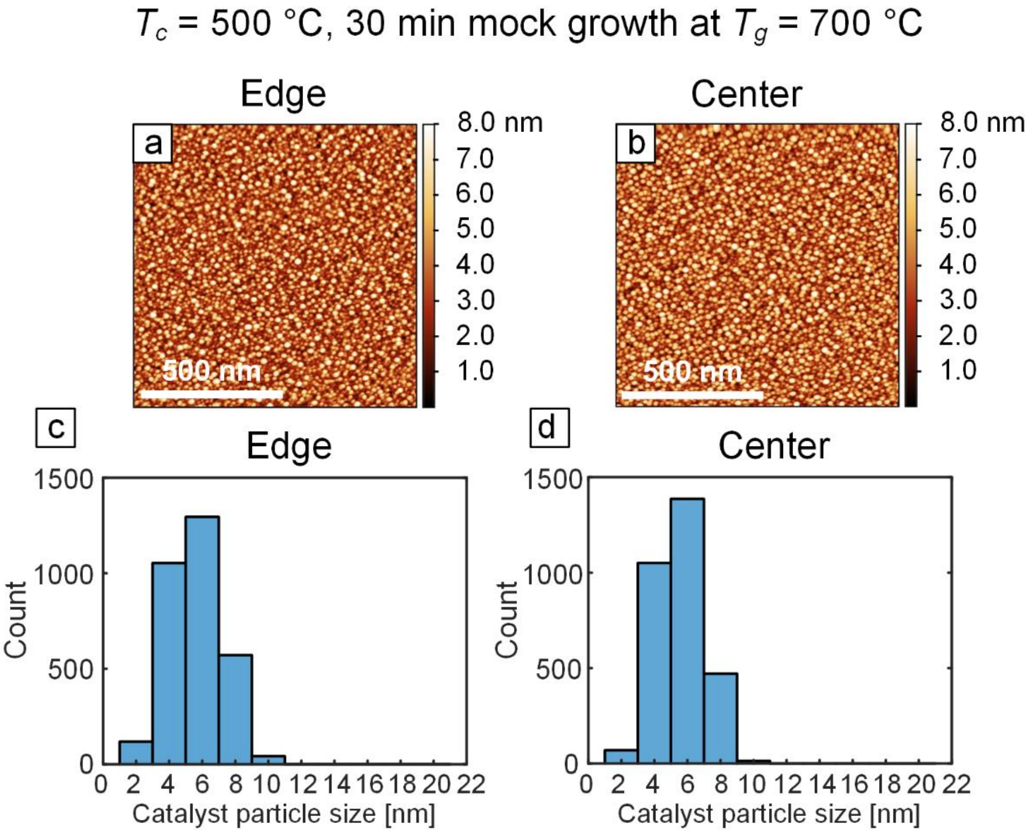

Fig. S5. Top view AFM images of (a) the edge and (b) center of catalyst chips annealed at T_c = 500 °C and underwent 30 minutes of mock growth at T_g = 700 °C. The bar charts in (c) and (d) illustrate the heigh distribution of catalyst nanoparticles, indicating insignificant differences in height between the edge and center of the sample.

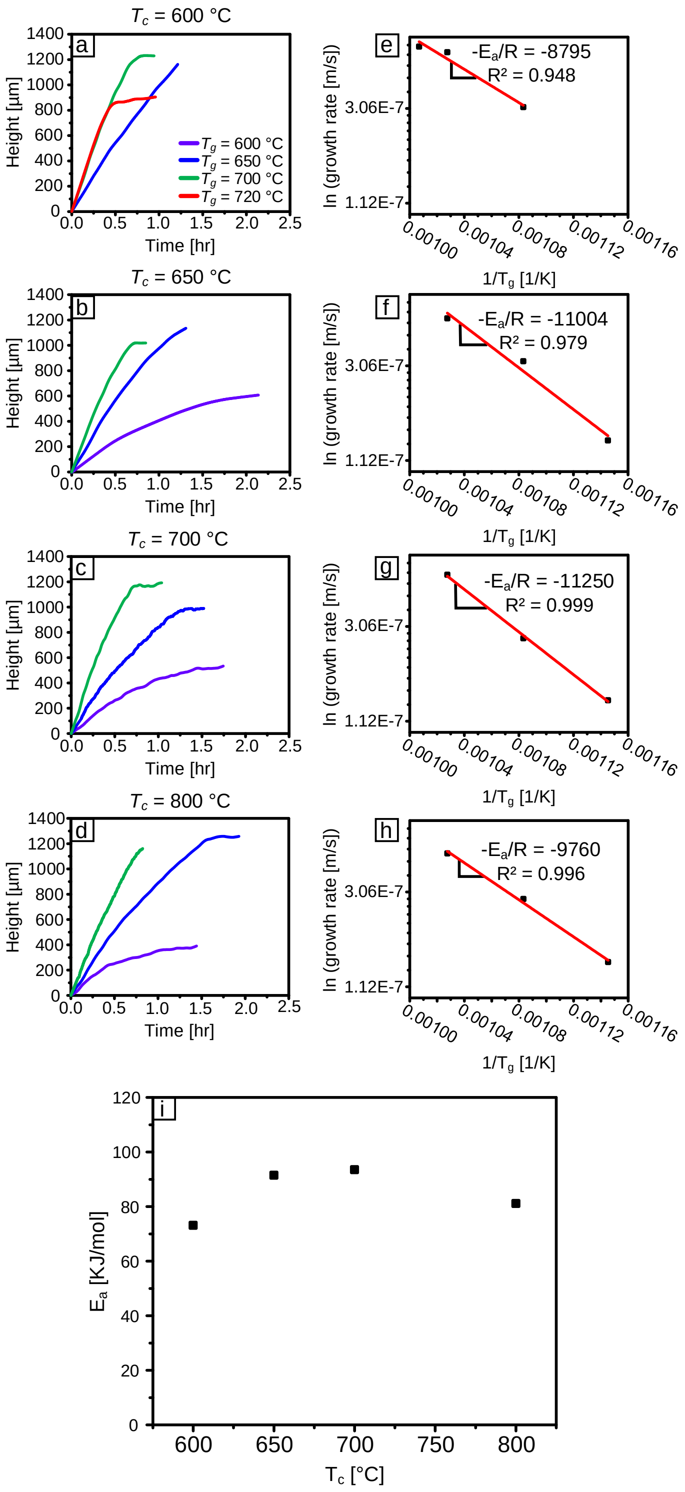

Fig. S6. (a-d) Height versus time curves depicting the growth kinetics of CNT forests at various T_g and T_c conditions. (e-h) Arrhenius plots demonstrate the relationship between ln(growth rate) and $1/T_g$, along with linear fits to determine activation energies. (i) Plot of activation energy values versus T_c, revealing relatively constant values with negligible dependence on T_c.

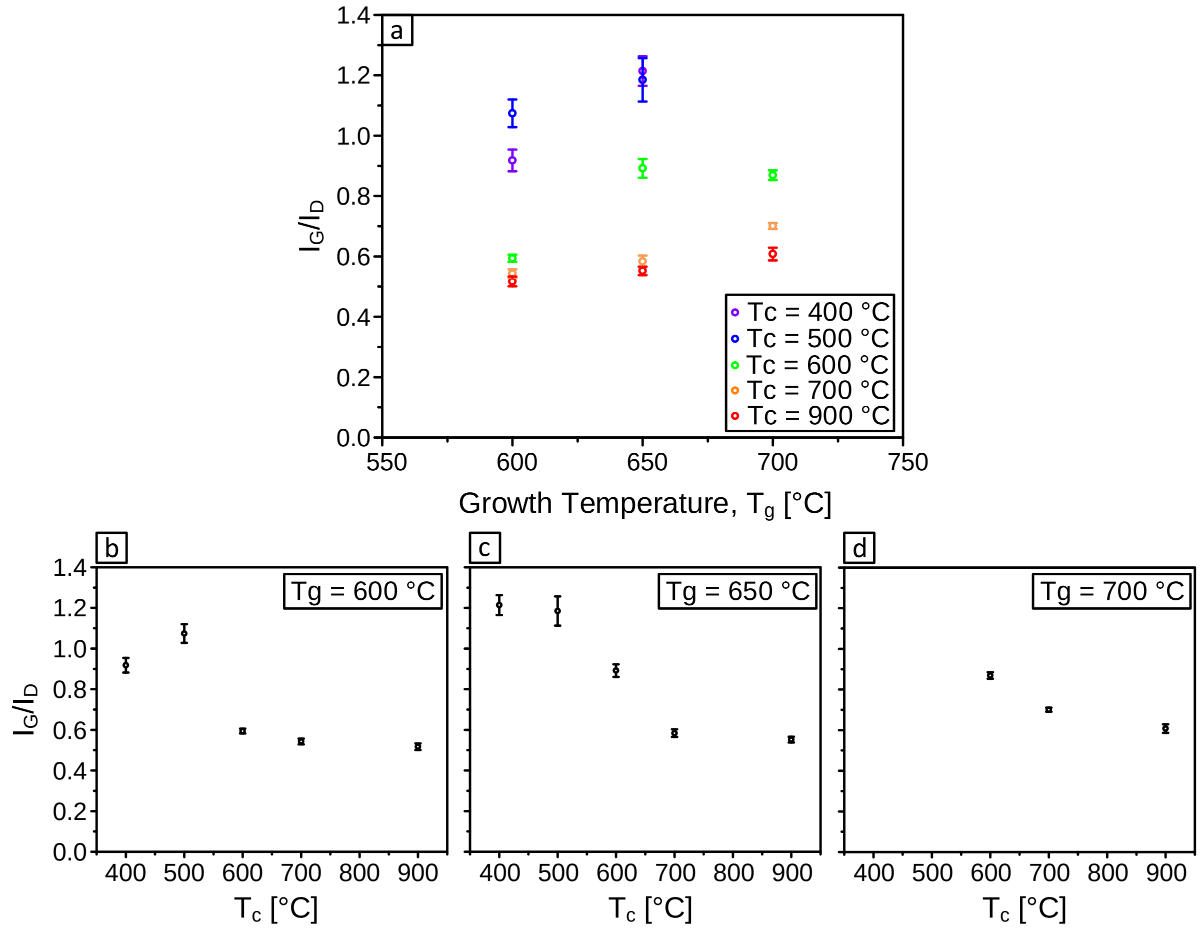

Fig. S7. (a) The ratios of I_G/I_D were determined by analyzing the top view Raman spectra of CNT forests grown at different T_c and T_g values. (Five different areas within each forest were measured to obtain error bars). (b-d) The dependence of I_G/I_D ratios on T_c for different T_g values shows a weakening trend at higher T_c.

Table S1. The areal number density of catalyst nanoparticles in the edge and center regions of each catalyst chip annealed at different T_c values.

T_c [°C]	Edge areal number density [nanoparticles/cm^2]	Center areal number density [nanoparticles/cm^2]
400	2.63×10^{11}	2.83×10^{11}
500	2.68×10^{11}	2.86×10^{11}
700	3.06×10^{11}	2.91×10^{11}
900	6.96×10^{10}	8.19×10^{10}
